# Umbilical cord blood metabolome differs in relation to delivery mode, birth order and sex, maternal diet and possibly future allergy development in rural children

**DOI:** 10.1371/journal.pone.0242978

**Published:** 2021-01-25

**Authors:** Alastair B. Ross, Malin Barman, Olle Hartvigsson, Anna-Carin Lundell, Otto Savolainen, Bill Hesselmar, Agnes E. Wold, Ann-Sofie Sandberg

**Affiliations:** 1 Department of Biology and Biological Engineering, Chalmers University of Technology, Gothenburg, Sweden; 2 Food and Biobased Products Group, AgResearch, Lincoln, New Zealand; 3 Dept. of Rheumatology and Inflammation Research, Institute of Medicine, Sahlgrenska Academy, University of Gothenburg, Gothenburg, Sweden; 4 Department of Paediatrics, Institute of Clinical Sciences, Sahlgrenska Academy, University of Gothenburg, Gothenburg, Sweden; 5 Clinical Bacteriology Section, Department of Infectious Diseases, University of Gothenburg, Gothenburg, Sweden; Copenhagen University Hospital Holbæk, DENMARK

## Abstract

Allergy is one of the most common diseases among young children yet all factors that affect development of allergy remain unclear. In a small cohort of 65 children living in the same rural area of south-west Sweden, we have previously found that maternal factors, including prenatal diet, affect childhood allergy risk, suggesting that *in utero* conditions may be important for allergy development. Here, we studied if metabolites in the umbilical cord blood of newborns may be related to development of childhood allergy, accounting for key perinatal factors such as mode of delivery, birth order and sex. Available umbilical cord blood plasma samples from 44 of the participants were analysed using gas chromatography-mass spectrometry metabolomics; allergy was diagnosed by specialised paediatricians at ages 18 months, 3 years and 8 years and included eczema, asthma, food allergy and allergic rhinoconjunctivitis. Nineteen cord blood metabolites were related to future allergy diagnosis though there was no clear pattern of up- or downregulation of metabolic pathways. In contrast, perinatal factors birth order, sex and mode of delivery affected several energy and biosynthetic pathways, including glutamate and aspartic acid—histidine metabolism (p = 0.004) and the tricarboxylic acid cycle (p = 0.006) for birth order; branched chain amino acid metabolism (p = 0.0009) and vitamin B_6_ metabolism (p = 0.01) for sex; and glyoxylate and dicarboxylic acid metabolism (p = 0.005) for mode of delivery. Maternal diet was also related to some of the metabolites associated with allergy. In conclusion, the cord blood metabolome includes individual metabolites that reflect lifestyle, microbial and other factors that may be associated with future allergy diagnosis, and also reflects temporally close events/factors. Larger studies are required to confirm these associations, and perinatal factors such as birth order or siblings must be considered in future cord-blood metabolome studies.

## Introduction

Childhood allergy is one of the most prevalent diseases among children in countries with a Western lifestyle [1]. Causes of allergy are incompletely understood. The hygiene hypothesis proposes that a reduced exposure to microbes during early life results in allergy development [2,3], but other lifestyle factors, such as maternal and early life diet, may contribute [4]. Children who grow up on small family farms are at much lower risk of developing allergy than other children living in rural areas [5–8]. Both exposures of the infant and the pregnant mother to the farming environment are thought to contribute to protection against allergy development in the child [9–11].

The FARMFLORA birth cohort was established to investigate factors of the farming environment that contribute to the protection of allergy in farm children. We recently showed that farming mothers ate more butter and less margarine during pregnancy and lactation compared to non-farming mothers [12], and that a high maternal margarine intake was associated with more allergy in the offspring [12]. We also found that high proportions of the long-chain omega (n)-3 polyunsaturated fatty acids (PUFAs) docosahexaenoic acid (DHA) in breast milk [13] and eicosapentaenoic acid (EPA) in the serum of new born infants [14] and of four-month old infants were associated with a decreased risk of allergy development, although this was unrelated to growing up on a farm [13].

Mechanisms behind why growing up on a farm may be protective against allergy have been suggested, including increased exposure to farm animals and their associated microorganisms, consumption of unpasteurized milk [11] and dietary differences as outlined in our earlier work [12]. A new avenue for deciphering the mechanisms behind disease development is to use metabolomics, the profiling of a wide range of metabolites in a sample [15]. Metabolomics not only detects molecules that represent endogenous metabolism, it also contains metabolites derived from food and the environment which together can provide important information about external factors which may trigger allergy [16]. Several studies have used metabolomics to better understand mechanisms behind certain types of childhood allergic diseases, especially asthma [17,18]. Several studies on newborn infants have used metabolomics to study factors associated with foetal exposures such as maternal smoking [19], and gestational diabetes [20,21], as well as child health outcomes, including birth weight [21], and preterm delivery [19]. Even though maternal and *in utero* factors are thought to be related to development of allergy, there is little known about whether the metabolome at birth could reflect future allergy risk. We hypothesised that metabolites in cord blood are influenced by foetal sex, mode of delivery, birth order and maternal diet during pregnancy and may predict allergy development.

In order to explore whether metabolites in the cord blood of newborn infants born in farm or non-farm environments could be related to future risk of ‘any allergy’ (i.e. the child have one or more of: food allergy, eczema, asthma or allergic rhinoconjunctivitis), we performed metabolomics on cord blood serum from a small observational study [12], We also investigated whether the metabolic profile of umbilical cord blood was associated with other factors associated with allergy development in the same cohort [12], such as diet, caesarean delivery, sex and being first-born.

## Materials and methods

### Subjects

The FARMFLORA birth-cohort study recruited 65 pregnant women at maternity clinics in the Skaraborg county in South-West Sweden between September 2005 and May 2008, 28 of whom lived on small dairy farms and 37 residing in the same rural areas, but not on farms. Participants from farming environments other than dairy farms, or urban areas were excluded. Final inclusion in the cohort occurred at delivery, when children born between gestational week 36 and 42 were included. Children were followed up regularly from birth until around eight years of age.

### Ethics statement

The study was conducted according to the relevant guidelines of the Declaration of Helsinki, and approved by the Regional Ethics Committee in Gothenburg (No. 363–05). Written informed consent was obtained from both parents. All participants were informed about their right to withdraw from the study at any point and to have their data removed.

### Power analysis

All available samples were used in this study, and no statistical power analysis was carried out due to no pre-defined hypotheses about which metabolites would be likely to change, nor available data in a similar cohort. This is in line with the exploratory nature of this metabolomics study.

### Sampling of umbilical cord blood

Cord blood was collected directly after delivery according to standard clinical routines at the hospital. The blood was allowed to clot for 30 min and centrifuged. Serum was removed, aliquoted and frozen. Frozen sera were stored at -80˚C until analyzed.

### Clinical examination

The children were examined clinically by pediatricians at 18 months, 3 years and around 8 years of age to diagnose food allergy, eczema, asthma and allergic rhinoconjunctivitis according to carefully standardized protocols as previously described [20,22,23]. All 65 children participated in the follow-up at 18 months, 63 children (97%) participated in the three year follow up and 48 of these children participated also in the 8-year follow up (76%, median age 8.3 years, range 6.5–9.4).

*Allergic rhinoconjuctivitis (ARC)* was defined as symptoms from eyes and/or nose upon exposure to pollen or animal dander, combined with demonstration of allergen-specific IgE to the corresponding inhalant allergen (Phadiatop, Phadia, Uppsala, Sweden).

*Asthma* was diagnosed at 18 months and 3 years of age based on ≥3 wheezing episodes combined either with a) eczema, allergic rhinoconjuctivitis or food allergy, or b) a response to leukotriene antagonists or inhaled glucocorticoids. At least one wheezing episode had to have occurred after 2 y ears of age for an asthma diagnosis at age 3 years. At 8 years of age asthma was defined as wheeze or heavy breathing in the last year together with a) bronchial obstruction reversible in response to β_2_-agonist, b) bronchial hyperresponsiveness on methacholine challenge or c) ongoing preventive asthma treatment with inhaled corticosteroids.

*Food allergy* was defined based on immediate or late-onset reactions after ingestion of a specific food that rapidly improved after allergen elimination. The diagnosis was supported by open food challenge tests and/or positive Fx5 Food Mix test (Phadia), followed by Immunocap tests (Phadia) for identification of the allergen.

*Atopic eczema* was diagnosed according to William’s criteria or based on at least three major Hanifin and Rajka criteria [24].

*Sensitization* against common foods (Fx5 Food Mix) and inhalant allergens (Phadiatop) were assessed by blood tests (Phadia, Uppsala, Sweden) [20].

Due to the low number of subjects who developed allergy over the follow-up period, all allergies were grouped together in the statistical analyses rather than separating into different types of allergy. Allergy diagnoses at the three follow up visits are outlined in **S1 Table**.

### Metabolomics

The metabolite profile of umbilical cord blood plasma was analysed by gas chromatography-mass spectrometry using a previously published method [25]. In brief, 100 μL of cord blood serum was extracted using 90% methanol with an internal standard mix to correct for variation in extraction and instrument response. Samples were derivatised using methoxymation and silylation and 1 μL was injected onto a Shimadzu GCMS8030 gas chromatograph-tandem mass spectrometer (GC-MS) [25]. Data were processed to identify compounds based on their retention index and spectrum against the Swedish Metabolomics Centre library. Only identified compounds were used for further data analysis.

### Maternal diet

Maternal diet data was collected using a semi-quantitive food frequency questionnaire (FFQ) adapted from the validated Northern Sweden 84-item questionnaire [26] extended with food items reflecting fat-containing foods [27]. The FFQ was sent out shortly after delivery and asked about intake frequencies the past year, hence covering the intake during the whole pregnancy. The methodology and results from the direct association between these assessments of maternal diet during pregnancy and allergy have been reported previously [12].

### Data analysis

The initial analysis of the cord blood metabolome focused on differences between children were born to parents living on dairy farms or not (the basis for the design of the study), and differences in cord blood metabolites between subsequently allergic and non-allergic children (controls) based on a diagnosis determined at 18 months, 3 years and 8 years of age. Children who were not allergic at 3 years or 8 years, but had been diagnosed as being allergic at an earlier time-point were not included in the non-allergic control group for those follow up ages. Statistical significance was tested using Mann-Whitney tests due to non-normal distribution of metabolite data. Further, the relation between cord metabolic profiles and three predefined factors that were previously determined to be associated with allergy were investigated, namely delivery mode (caesarean or vaginal) [28], being first born [2], and being of male sex [29]. The relationship between metabolite pattern and allergy diagnosis was also adjusted for these three factors using logistic regression. Sensitivity analyses were performed where difference in cord blood metabolites between allergic and non-allergic children, at 18 months and 3 years, were analyzed in the non-farm group only by excluding all children in the farm group from the analysis. Statistical analyses were carried out using R version 3.5.1.

As we have previously found maternal diet during pregnancy to be associated with childhood allergy in this cohort [12], maternal food frequency scores were correlated with umbilical cord metabolites that were found to be associated with future allergy development at any timepoint using Spearman’s rank correlation. Correlations >0.4 or <-0.4 are reported. Due to the low number of subjects, maternal diet was not included as a factor in the logistic regression models.

Principal Components Analysis (PCA) was used to identify any outliers due to analytical issues (Simca 15, Umetrics AB, Sweden).

The potential effects of the metabolite differences on metabolic pathway upregulation was tested using the Pathway Analysis tool in MetaboAnalyst [30]. Separate analyses for allergy at 18 months and 3 years and 8 years of age, exposure to farming conditions, mode of delivery, having siblings or not and sex were carried out. All identified cord blood metabolites with corresponding Kyoto Encyclopaedia of Genes and Genomes (KEGG) metabolite identification numbers were included in this analysis (133 out of 155 metabolites), and data were log-transformed prior to analysis and mean centred prior to analysis. The data were matched to the KEGG human metabolite pathway library (Kyoto Encyclopaedia of Genes and Genomes, www.genome.jp).

Differences between conditions for metabolites, and pathway enrichment were considered significant at P<0.05, with no correction for multiple testing in line with the exploratory nature of this study.

## Results

Characteristics of the 44 included families are shown in Table 1. The mean maternal age of the women were 33 years (range 21–42 years). The infants were born between gestational week 36 and 42 with a mean of 39 weeks. The mean birth weight was 3512 grams (range 2730–4435). One infant was born large for gestational age (LGA) while no infants were born small for gestational age (SGA) according to ultrasound-based growth curves [31]. Four infants were born preterm, i.e. before 37 full weeks of gestation. Around half of the infants were of male sex, 6 were born with caesarean section, 26 had older siblings and 4 were part of a twin pair (Table 1). The mothers of the children in this cohort were not undergoing any specialist medical care during their pregnancy so maternal health has not been included as a factor in subsequent analyses.

**Table 1:** Demographic data of the 44 included mother/new-born dyads.

	Mean or n	SD(%)
Maternal age	33	(4.3)
Gestational age, weeks	39	(1.6)
Gestational age, days	278	(11)
Birth weight	3512	(403)
SGA	0	(0%)
LGA	1	(2%)
Preterm birth	4	(10%)
Male sex	21	(48%)
Twin pairs	2	(5%)
Caesarean section	6	(14%)
Older siblings	26	(59%)

Small for gestational age (SGA) and large for gestational age (LGA) status according to ultrasound-based growth curves [31]. Preterm birth is defined as delivery before 37 full weeks of gestation.

A total of 155 metabolites were identified in cord blood plasma after matching for retention index and mass spectra, and after visual inspection of the mass spectrum and chromatogram for the aligned peak result. No trends due to analysis were observed, and nor was there any clear grouping for allergy, farming, delivery mode, birth order, siblings or sex with PCA (**S1 Fig**).

### Effect of farm residence

Twelve metabolites were associated with perinatal exposure to a farming environment, but none of them very strongly (**Table 2**). Infants born to mothers living on dairy farms had higher levels of lactose, n-acetyl-l-cysteine, cellobiose, lactulose, pyruvic acid, glycolic acid, rhamnose, aconitric acid and lyxose while infants born to non-farming mothers had higher levels of anhydro-d-glucitol, ursodeoxycholic acid and hydroxybutyric acid. There were no metabolic pathways impacted by farm vs non-farm residence.

**Table 2:** Metabolic differences in umbilical cord blood between farm and non-farm infants.

	*Farm*, *n = 21*		*Non-farm*, *n = 23*			
	Median	IQR	Median	IQR	P-value	Fold change
**Anhydro-D-gluticol**	52506	23677	62409	27438	0.013	-0.42
**Lactose**	4998	3850	2706	2823	0.013	0.55
**N-acetyl-L-cysteine**	447895	340228	273604	149632	0.020	0.72
**Cellobiose**	4751	3484	2602	2283	0.028	0.51
**Ursodeoxycholic acid**	29686	24368	55408	39445	0.034	-0.64
**Lactulose**	5852	3635	3126	2523	0.040	0.47
**Pyruvic acid**	123734	48985	107303	40785	0.040	0.41
**Glycolic acid**	37458760	10366464	32018982	7180247	0.042	0.27
**Rhamnose**	2696570	1824822	1747505	943766	0.042	0.57
**Aconitic acid**	138590	64230	111944	55014	0.045	0.28
**Lyxose**	4019	2054	3274	1609	0.045	0.37
**Hydroxybutyric acid**	18851	21051	21709	28178	0.048	-0.64

Data are GC-MS peak areas. Differences between farm and non-farm infants were analysed with Mann-Whitney U-test.

### The effect of delivery mode

The three predefined factors that are known to be associated with allergy, i.e. mode of delivery (caesarean or vaginal delivery) [28], whether the child was the first born or had siblings [2], and sex [29], were all found to be strongly associated with metabolic differences. Fourteen metabolites differed between children delivered vaginally and through caesarean section, covering several types of metabolites including amino acids, carbohydrates and one-carbon metabolism (**Table 3; S2 Fig**). Pathway analysis of the identified cord metabolites found that glyoxylate and dicarboxylic acid pathways were significantly altered based on whether children were born by vaginal or caesarean section delivery (p = 0.005). Of the six caesarean sections, one was acute, four were planned while no information was available for one.

**Table 3:** Significant metabolic differences between mode of delivery (caesarean section or vaginal delivery).

	Caesarean section, n = 6		Vaginal delivery, n = 38			
	Median	IQR	Median	IQR	p value	Fold change
**Lyxose**	2345	937	3669	1852	0.0021	-0.83
**Quinic acid**	43781	19374	79624	30289	0.0130	-0.66
**Uridine**	30185	8908	25170	9019	0.0145	0.39
**Ribose-5-phosphate**	19612	5896	11732	7992	0.0222	0.51
**Alanine**	44880	17508	58163	27489	0.0246	-0.52
**Fructose**	4705	1965	11450	17622	0.0271	-1.80
**Sorbose**	4403	2143	11344	18241	0.0271	-1.81
**Creatinine**	44201	29212	29821	11690	0.0299	0.54
**Homocysteine**	24301	13183	12929	9345	0.0299	0.60
**Nigerose**	2463	1799	4496	4064	0.0299	-0.96
**Valine**	275398	14795	338292	80070	0.0299	-0.27
**Homoserine**	25773	13377	35253	16044	0.0361	-0.53
**Glucosamine**	173120	262624	373811	323590	0.0434	-0.94
**Isoleucine**	73896	21988	87316	28365	0.0434	-0.40

Data are GC-MS peak areas. Differences between the two groups were analysed with Mann-Whitney U-test.

### The effect of birth order

The effect of being first born or having older siblings was strongly imprinted in the umbilical cord metabolome, with 25 metabolites differing between these two groups (**Table 4; S3 Fig**). Birth order affected several metabolic pathways: glutamate and aspartic acid–histidine metabolism (p = 0.004), tricarboxylic acid (TCA) cycle (p = 0.006), fructose and mannose metabolism (p = 0.01) and butanoate metabolism (p = 0.01).

**Table 4:** Significant metabolic differences between children with or without siblings.

	Have older siblings, n = 26		No older siblings, n = 18			
	Median	IQR	Median	IQR	p value	Fold change
**Oxalic acid**	159634	46356	215444	91995	0.00003	-0.69
**Citric acid**	11302	4506	18922	8789	0.00124	-0.57
**Inositol**	46903	22119	34617	14392	0.00124	0.71
**N-acetyl glucosamine**	1707288	821658	1406169	531941	0.00214	0.72
**Phenylpyruvic acid**	64297	16030	79925	20230	0.00214	-0.32
**Sorbitol**	3249	2773	5449	5676	0.00256	-0.87
**Lactic acid**	298432	51095	366236	90623	0.00279	-0.23
**Isoerythritol**	375786	133672	498367	195137	0.00424	-0.35
**α-tocopherol**	161923	91978	245157	75151	0.00738	-0.28
**Glutamic acid**	51698	26943	33843	11331	0.00796	0.46
**N-acetylmannosamine**	166880	67235	132443	54499	0.00796	0.53
**Fumaric acid**	223664	101344	305973	141690	0.01428	-0.41
**Xylose**	3313	1354	4395	2440	0.01428	-0.40
**Ornithine**	33095	16235	20638	14835	0.01532	0.49
**Malic acid**	2967379	1341621	3740148	1756233	0.01641	-0.41
**Nigerose**	3604	2447	4951	4616	0.01758	-0.62
**Cellobiose**	2795	3111	4665	4679	0.01881	-0.67
**N-acetylornithine**	74127	54777	107498	57345	0.02012	-0.55
**Allothreonine**	79426	28318	71611	17009	0.02297	0.26
**Isocitric acid**	35225	14285	43186	15557	0.02297	-0.27
**Aspartic acid**	1654723	604487	1383135	267027	0.02787	0.25
**Lactose**	2651	3098	4884	4441	0.02970	-0.62
**Xylitol**	19956	14046	33050	12069	0.03161	-0.46
**Lactulose**	3089	3138	5899	4173	0.04284	-0.57
**Phenylalanine**	1464103	549524	1372503	293543	0.04284	0.22
**Arabinose**	74523	39838	98879	37775	0.04544	-0.43

Data are GC-MS peak areas. Differences between the two groups were analysed with Mann-Whitney U-test.

### The effect of sex

Ten metabolites differed between male and female infants (**Table 5; S4 Fig**). Notably, all metabolites that differed between sexes were higher in girls except for erythritol and 2-oxoisocaproic acid.

**Table 5:** Significant metabolic differences between male and female sex.

	Male, n = 21		Female, n = 23			
	Median	IQR	Median	IQR	p value	Fold change
**Amiloride**	39878	5597	45074	15684	0.009	-0.28
**Leucine**	31319	9779	36673	23121	0.010	-0.50
**Isoleucine**	80114	26489	98224	50301	0.013	-0.40
**Galactitol**	1029	2346	3146	4555	0.031	-1.32
**Hydroxybutyric acid**	17053	21402	24599	19108	0.032	-0.66
**Uric acid**	51282	47882	65892	93350	0.033	-0.90
**Erythritol**	2221990	1257668	1595929	926665	0.036	0.54
**Sucrose**	8834	5910	13690	20589	0.040	-1.21
**Mannose**	3416	6329	5442	15173	0.042	-0.05
**2-oxoisocaproic acid**	27838	14608	22864	12665	0.045	0.28

Data are GC-MS peak areas. Differences between the two groups were analysed with Mann-Whitney U-test.

The sex of the children was reflected in differences between several pathways; branched chain amino acid metabolism (p = 0.0009), vitamin B_6_ metabolism (p = 0.01), taurine and hypotaurine metabolism (p = 0.01), pyruvate metabolism (p = 0 .017), terpenoid backbone biosynthesis (p = 0.017), cysteine and methionine metabolism (p = 0.018), glyoxylate and dicarboxylate metabolism (p = 0.024), and nicotinate and nicotinamide metabolism (p = 0.026).

### Prediction of future allergy by cord blood metabolic pattern

Eight metabolites were associated with allergy development when all follow up timepoints were considered (“allergy at any time-point”, **Table 6**). However, of these eight, only ursodeoxycholic acid was predictive for allergy development at any one of the individual time points, being higher in infants diagnosed with allergy at 18 months of age. Cord blood metabolites that were associated with future allergy diagnosis differed according to the time point at which allergy was diagnosed (**Table 6 and S5–S7 Figs**) and no single metabolite was associated with allergy at all time points, though α-ketoglutaric acid was lower in the cord blood of children who would go on to be diagnosed with allergy at 18 months and 3 years. Notably, there was a stronger association between cord blood metabolite composition and allergy at age 8 years, than with allergy at the earlier time points (Table 6). The sample size was too small to allow further investigation based on the type of allergy diagnosis. Adjustment for being first-born, male sex and caesarean delivery attenuated approximately half of the unadjusted analysis differences between allergic and non-allergic children (**Table 6**).

**Table 6:** Significant differences in cord blood metabolites between subsequently allergic and non-allergic children based on diagnosis of allergy at different ages.

***Allergy at any time point***	**Allergic, n = 14**		**Not allergic, n = 30**		**p values**					**Fold change**
					**unadjusted**	Adjusted*				
	**Median**	**IQR**	**Median**	**IQR**		**BO S DM**	**BO DM**	**BO S**	**S DM**	
**Opthalmic acid**	23843	14351	36824	34405	0.016	0.048	0.044	0.054	0.049	-0.522
**Ursodeoxycholic acid**	55213	47987	31957	34161	0.019	0.010	0.008	0.018	0.010	0.726
**δ-tocopherol**	29378	21603	16356	13392	0.027	0.027	0.026	0.034	0.027	0.622
**Glyceric acid**	1824	872	2633	1697	0.034	0.076	0.069	0.088	0.080	-0.411
**Lactose**	2682	2612	4002	4051	0.035	0.105	0.108	0.082	0.119	-0.690
**Cellobiose**	2558	2474	3640	4200	0.038	0.108	0.113	0.084	0.124	-0.701
**Sorbitol**	4245	3702	3950	7878	0.040	0.111	0.141	0.094	0.125	-0.798
**Nigerose**	3564	2321	4649	3978	0.047	0.127	0.141	0.097	0.145	-0.607
*18 months*	**Allergic, n = 10**		**Non-allergic, n = 34**							
**Ursodeoxycholic acid**	57662	34659	32062	33350	0.021	0.012	0.029	0.010	0.013	0.674
**Pyroglutamic acid**	607144	108081	664308	116454	0.026	0.034	0.031	0.031	0.042	-0.148
**α-ketoglutaric acid**	25078	2136	31390	9669	0.041	0.112	0.080	0.087	0.135	-0.245
*3 years*	**Allergic, n = 6**		**Non-allergic, n = 31**							
**Oxoisocaproic acid**	16762	3623	27249	13368	0.019	0.053	0.049	0.040	0.062	-0.549
**Uridine**	31445	5643	25161	7826	0.022	0.042	0.045	0.028	0.040	0.423
**Benzoic acid**	30115	3808	35220	7458	0.025	0.109	0.106	0.093	0.108	-0.292
**Putrescine**	12761	13004	9209	4980	0.035	0.041	0.039	0.034	0.044	0.804
**α-ketoglutaric acid**	24001	1716	31714	9364	0.044	0.145	0.109	0.111	0.146	-0.303
**Isocitric acid**	30102	11344	42209	15486	0.048	0.037	0.050	0.034	0.045	-0.360
*8 years*	**Allergic, n = 7**		**Non-allergic, n = 24**							
**E-Octadecenoic acid**	3599571	432707	2700887	889046	0.003	0.023	0.016	0.044	0.017	0.391
**Z-Octadecenoic acid**	3213916	306038	2602898	748100	0.011	0.051	0.046	0.127	0.045	0.231
**Aspartic acid**	1284643	246623	1513347	420257	0.016	0.031	0.040	0.032	0.040	-0.318
**Cholic acid**	1420	196	857	498	0.022	0.141	0.095	0.122	0.139	0.544
**Hydroxyphenylacetic acid**	11028	5790	7579	4389	0.033	0.099	0.161	0.070	0.097	0.665
**Hexadecanoic acid**	225387	38680	194448	39945	0.041	0.030	0.031	0.049	0.028	0.216
**N-acetylornithine**	109336	51788	72155	44892	0.041	0.057	0.070	0.066	0.068	0.611
**Uric acid**	78720	115274	46950	33403	0.041	0.224	0.133	0.128	0.230	1.113

Data are GC-MS peak areas. Differences between the two groups were analysed with Mann-Whitney U-test.

* Adjusted for: Birth Order (BO), Sex (S) and Delivery mode (DM).

Some of the metabolites that were associated with allergy status were also associated with a farming environment. For example, ursodeoxycholic was lower in infants from farming families than from non-farming families (Table 2) and higher in allergic compared to non-allergic infants at 18 months of age as well as being allergic at any time point during follow up (Table 6). Conversely, disaccharides lactose and cellobiose were higher in the cord blood of children born to farmers (Table 2), and lower in children who developed allergy at any point during follow up (Table 6). Accordingly, allergy was much more prevalent in non-farming than farming families at the two first diagnostic time points [12] and for the 18 months and 3 years follow ups, growing up on a farm or not is a major confounding factor for the allergy analysis.

To account for the difference in prevalence of allergy in the non-farming and farming families, we performed sensitivity analyses on difference in cord blood metabolites between allergic and non-allergic children, at 18 months and 3 years, in the non-farm group only (**Table 7**). None of the three metabolites that were associated with allergy at 18 months of age (ursodeoxycholic acid, pyroglutamic acid, alpha-ketoglutaric acid) were associated with allergy when only children in the non-farm group were included in the analyses between allergic and non-allergic individuals. Instead four other metabolites were significantly different between allergic and non-allergic individuals (δ-tocopherol, dodecanoic acid, glucose and myristoleic acid). However, when examining allergy at 3 years of age three of the six metabolites that differed between allergic and non-allergic individuals in the whole cohort were still significantly different when looking only in the non-farm group:, uridine, benzoic acid and alpha-ketoglutaric acid. At 8 years of age, the distribution of allergy in the farm and non-farm group did not differ statistically (p = 0.43) with 7 allergic children in the non-farm group and 3 allergic children in the farm group. Hence, no sensitivity analyses based on farming environment were performed for allergy at this age.

**Table 7:** Significant metabolic differences between allergic and non-allergic children based on diagnosis at 18 months and 3 years in the non-farm group only.

	Allergic, n = 8		Non-allergic, n = 15			
*18 months*	Median	IQR	Median	IQR	p value	Fold change
**δ-tocopherol**	18667	18009	17498	17895	0.026	0.62
**Dodecanoic acid**	56684	32315	78308	41385	0.034	-0.09
**Glucose-6-phosphate**	1432	816	1867	1458	0.045	-0.87
**Myristoleic acid**	193299	108112	229996	182648	0.047	-0.58
*3 years*	Allergic, n = 5		Non-allergic, n = 14			
**α-ketoglutaric acid**	26937	10347	29692	9288	0.026	-0.36
**Uridine**	25733	4878	23070	6345	0.026	0.45
**Monostearoylglycerol**	33446	13958	44943	106907	0.044	-0.48
**Adenosine**	14825	13161	20101	12181	0.044	0.76
**Asymmetrical-N-N-dimethylarginine**	276868	114167	269977	158079	0.044	-0.37
**Benzoic acid**	31213	2741	36317	12814	0.044	-0.39
**Glucose-6-phosphate**	1392	718	1884	1239	0.044	-1.05

Data are GC-MS peak areas. Differences between the two groups were analysed with Mann-Whitney U-test. Statistical analyses were not adjusted for any covariables due to the low number of participants when only including non-farm children.

Pathway analysis did not find any significant differences between cord blood metabolome at birth in allergic and non-allergic children at 18 months, 3 years or 8 years of age, suggesting that of the metabolites that were mapped in metabolic pathways, those that differed did not represent a concerted difference in metabolic status and instead likely reflect the impact of genetic, lifestyle and other environmental differences.

To probe this, we tested the association between maternal diet and metabolites associated with allergy in the overall cohort. Of the 23 metabolites that were associated with allergy overall or any time point, 19 were also correlated with one or more foods in the maternal diet during pregnancy (S2 Table).

## Discussion

In this small, but well controlled study we found that metabolites in umbilical cord blood were strongly associated both with delivery mode, sex and birth order, with potentially important impacts on metabolic pathways. Cord blood metabolites were also associated with prenatal exposure to maternal diet and a farming environment and later allergy development but were not indicative of consistent systemic metabolic effects.

Our aim was to identify pre- and peri-natal factors associated with subsequent allergy development, but we did not find any relationship with perturbation of metabolic pathways, though both low statistical power and scattered metabolite coverage may have hindered finding differences in biochemical pathways related to future allergy.

The strongest relationships with the cord blood metabolome were events that were in close temporal proximity to the sample being collected (delivery mode) or of fundamental biological importance (sex and birth order). While sex imprinting on the metabolome of infants has been frequently described [32,33], as has the difference in delivery mode [34–36], we were surprised to find that birth order (i.e. first born or having older siblings) was also reflected in the metabolome. Having older siblings or not impacted on several related metabolic pathways, with the TCA cycle, butanoate metabolism, aspartic acid and glutamate metabolism all being linked, especially through fatty acid metabolism and acetyl-CoA. Possible reasons are speculative, though practical aspects such as the mother having already experienced giving birth leading to a different level of psychological and physiological preparedness, or the need to look after another child during pregnancy may play a role. Time may also be a factor, and although time from rupture of membranes to delivery was not recorded in this study, in general first deliveries take longer than subsequent deliveries [37]. In support of the hypothesis that use of energy, length of delivery and overall effort being greater for first delivery is that the concentration of energy metabolism end product lactic acid was higher in cord blood of children with no siblings, as were TCA-cycle metabolites. Further, lactic acid, an end product of muscle energy metabolism, was borderline lower in cord blood after caesarean delivery (p = 0.061), suggesting that muscular contractions do impact on the cord blood metabolome and is supported by earlier work comparing delivery mode using metabolomics [34,35]. As a crude measure of the relative importance of having siblings versus other factors, the number of metabolites related to having siblings or not, was more than twice that of either sex or delivery mode (26 compared to 12 and 14 respectively). Most metabolites that differed between children with siblings or no siblings were higher in those with no siblings (20 out of 26).

Mode of delivery also led to several differences in umbilical cord metabolites, notably a higher concentration of homocysteine in caesarean delivered children compared to vaginally delivered children. Homocysteine, an intermediate of one carbon metabolism and DNA methylation, has been linked to birth complications [38], and has previously been found to be higher in women having caesarean sections under general anaesthesia [39]. The elevation of homocysteine in the cord blood of caesarean delivered infants may reflect differences in requirements for methyl-groups and increased use of methyl-donors and related enzyme cofactors such as folate and vitamin B_12_. We did not have information about the time between rupture of membranes and delivery, and this may also be informative in future studies if delivery mode has an impact on the cord blood metabolome. There did not appear to be any clear metabolic connection between factors associated with delivery mode, siblings or sex that could be clearly linked to metabolic factors that were related to allergy risk.

The metabolites that differed between children who went on to develop allergy compared to those who did not covered a wide range of compound classes, though notably included several that are related to lipid and energy metabolism including bile acids (ursodeoxycholic acid and cholic acid), fatty acids (octadecenoic acids 9Z and 9E, eicosapentaenoic acid, and hexadecenoic acid), amino acid derivatives (pyroglutamic acid, 2-oxoisocaproic acid, aspartic acid, acetylornithine and putrescine) and Krebs Cycle intermediates (α-ketoglutaric acid and isocitric acid). Earlier work in this cohort on both the fatty acid profile of the infants [13] and maternal intake of dietary fat [12] have found that both have an impact on allergy risk. The finding that bile acids were elevated in the cord blood of infants that went on to develop allergy supports the role of dietary lipids in allergy development in this cohort. Bile acids have been associated with immune regulation and allergy [40,41], though given the potential confounding from different fat intakes and endogenous fatty acid profiles, it is not possible to establish if there is a causative relationship between elevated bile acids and allergy in this study.

The metabolites associated with allergy development differed when comparing allergy status at different ages. Symptomatic allergy is variable with age, often seen with children ‘growing out of’ allergy, while more severe and longer-term allergy is observed in older children, reflected in the eight year allergy diagnosis follow up, a phenomenon called ‘the atopic march’ [42]. Only three umbilical cord metabolites differed between children who were diagnosed as being allergic at 18 months, while increasing numbers were different in relation to allergy diagnosis at the later follow up visits (six and eleven metabolites at 3 years and 8 years respectively). Only α-ketoglutaric acid was associated with allergy at two time points (18 months and 3 years). Metabolites related to protein breakdown and central energy metabolism were related to 3 year diagnosis, while several lipids differed between allergic and non-allergic children at 8 years old, which were not at all related to allergy diagnosis at 18 months and 3 years. Metabolites related to inflammatory pathways were not covered in this method, so it is not possible to state whether the umbilical cord concentrations of metabolites in these pathways with a clear relationship to allergy were in anyway related to future allergy. The possibility that later life allergy is imprinted already at birth is intriguing, and this would support the idea that conditions around *in utero* development and birth, along with genetic and general lifestyle factors may be associated with long-term allergy development. However, there were no overall effects on metabolic pathways detected, while such effects were detected for factors that would be expected to impact on the metabolome at the time of sample collection (e.g. sex, delivery mode). This also raises the possibility that the metabolites related to future allergy instead reflect lifestyle and familial factors that are related to future allergy risk, rather than being directly related biochemically to future allergy development. We investigated this in a further analysis on the association between maternal diet and cord blood metabolites related to allergy.

The role of maternal diet during pregnancy in the development of allergy is of great interest as it presents a potential modifiable factor in allergy risk [12,43]. In the current study we found that the metabolites that were associated with allergy risk were associated with many, and diverse, dietary factors. We did not model these dietary factors within the metabolite-allergy model due to the low overall number of subjects. We have earlier found that maternal diet during pregnancy influenced allergy in this cohort, with margarine intake being associated with higher allergy risk [22]. It was notable that many of the foods associated with allergy-related metabolites were also related to dietary fats. Higher intake of margarine or soya oil overall or in cooking was associated with metabolites that were predictive of allergy at 3 and 8 years (E- and Z-octadecanoic acid, hydroxyphenylacetic acid and uric acid), while the use of olive oil, rapeseed oil and butter on sandwiches were negatively associated with metabolites that predicted allergy at 3 and 8 years (hydroxyphenylacetic acid). In the case of 2-oxoisocaproic acid, a metabolite associated with lower risk of allergy, higher concentrations were measured with higher butter intake, and lower concentrations with higher margarine intake. These findings further support the importance of choice of dietary fat in the maternal diet during pregnancy for future allergy risk in this population. Due to the small size of this cohort we have not sought to interpret the potential-diet-cord metabolome-allergy interaction, only to highlight that there are many relationships between maternal diet and the cord blood metabolome and that this should be of future interest.

The children included in this study were from the FARMFLORA study, a small but well-controlled birth cohort following families living on dairy farms and families living in the same rural area but not on farms, with the aim to determine what in the farming environment could be responsible for the apparent protection against developing childhood allergy. We have previously found that eating habits among farmers and their infants differ from the families that do not live on farms [12,13,44]. For example pregnant mothers not living on dairy farms eat more margarine which was also associated with allergy in their infants [12]. Sensitivity analysis when non-farm raised children were removed from the model suggested that the differences between cord blood metabolites and allergy diagnoses at 18 months and 3 years could not be disentangled from whether children were raised on farms or not. This may further explain the difference between metabolites associated with allergy at the two first follow ups and the 8 year follow up, as the impact of maternal environment plays less of a role as the children grow older. Other metabolites, including uridine, benzoic acid and α-ketoglutaric acid were consistent in their association with allergy in both non-farm children and the entire cohort.

Only three metabolites were associated with both farming environment and allergy; ursodeoxycholic acid, lactose and cellobiose. Ursodeoxycholic acid, a bile acid, suggests a difference in lipid metabolism which matches earlier results on differences in maternal fat intake [12], and infant blood fatty acid composition [13]. The two disaccharides lactose and cellobiose may also be related to differences in dietary intake, with lactose being found in substantial quantities in milk, and cellobiose potentially coming from plant-based foods. In both cases it would be expected that most lactose and cellobiose is broken down during maternal absorption, though both compounds have been reported previously as being detected in plasma. As bile acids interact with the intestinal microbiota and are modified by conjugation [45], and both lactose and cellobiose represent fermentable substrate that has avoided breakdown in the large intestine, it is possible that these plasma metabolite differences are also indicative of a diet-environment-microbiome interaction playing a role in allergy development, though more detailed analyses on both faecal samples and microbe-related metabolites will be needed to prove this.

There are a number of weaknesses in this study and approach used which should be accounted for when interpreting the data. We did not use correction for multiple testing, which increases the likelihood of false positive results, due to the low number of subjects in the study. While this restricts the possible statistical power and generalisability of the outcomes, it has the advantage of allowing the cohort to be geographically well defined, and to measure many exposures in relation to doctor diagnosed allergy outcomes in a prospective setting and starting during pregnancy. The rate of allergy among those children in the study living on dairy farms could be a confounding factor in this study as the markers may reflect lifestyle rather than allergy risk. However all children in the study lived in the same rural environment, including while *in utero*, so while exposure to microorganisms from animals would be expected to be higher among children born to dairy farmers, other exposures such as weather and pollen are likely to be similar. Sensitivity analysis suggests that growing up on a farm or not is an important factor for metabolites related to childhood allergy in this study. Not all possible factors that have previously been related to allergy were controlled for, for example season of birth [46,47] or maternal physical activity and other lifestyle factors [48]. This study also had a moderately high loss to follow up at 3 and 8 years, representing 18% of the total children diagnosed with allergy. This number of participants dropping out of the study is not unexpected, especially at 8 years where families had moved from the region where the study was located. This does reduce the statistical power at the 8 year follow up, and the number of allergic children is reduced from 15 at 18 months to 11 at 3 years and 10 at 8 years. However this also follows the expected decline in allergy prevalence as children grow older. Although we have not stratified this analysis by type of allergy, it is also notable that eczema is the most prevalent allergy diagnosis at 18 months (73% of diagnoses including eczema, declining to 50% at 8 years). Of the four lost to follow up at 8 years, allergy diagnoses at earlier time points included eczema, asthma, food allergy and allergic rhinoconjunctivitis, suggesting that there was no impact of the type of diagnosis on participants dropping out of the study. The age of the children at the third follow up varied (range 6.5–9.4 years). This may confound comparisons of allergy diagnoses at either end of this range which spans 3 years. This age range is due to the practicalities of getting the children to the third follow up visit with the same paediatrician, meaning that it was not possible to have all children at exactly the same age when this was done. When detecting the impact of delivery mode on the cord blood metabolome, we have grouped together all types of caesarean deliveries, which themselves can differ widely, especially between elective and emergency caesarean delivery. With only six cases of caesarean delivery we did not have sufficient statistical power to further divide the cohort by type of caesarean delivery. Similarly, there was only one child who was born large for gestational age, and two twin pairs and four children born pre-term in the cohort, so no sub-analyses were carried out for these factors.

Analytical methods are crucial to account for in metabolomics as the type of approach used dictates what metabolites are included in the final analysis. In this study we have used GC-MS for metabolomics, an instrument which has the advantage of allowing rapid identification of compounds based on mass spectral and retention index matching, but does not have the breadth of metabolite coverage of liquid chromatography-mass spectrometry when several different chromatographic modes are used. Not all identifications made using spectral and retention time matching could be confirmed, and the identity of one metabolite strongly associated with allergy at eight years could not be confirmed based on spectral matching and hence not included in the overall analysis. Additional metabolomics methods should be used to get wider coverage of the metabolome to discover other metabolites that are associated with allergy development.

## Conclusions

Results from this work suggests that the umbilical cord blood metabolome is strongly influenced by sex, delivery mode and birth order, with apparent impacts on several biochemical pathways. There may also be imprinting of future allergy risk, though consistent changes related to mapped biochemical pathways were not evident and these metabolites may instead reflect lifestyle and familial factors that are in turn related to allergy risk. Maternal diet during pregnancy appears to be reflected in cord blood metabolites associated with allergy, supporting the hypothesis that maternal lifestyle may have an impact on future childhood allergy. These results underline that the metabolome is highly responsive to a myriad of factors, and that important but often overlooked covariables in health research such as siblings or delivery mode need to be accounted for in studies analysing samples collected at birth. Further work needs to be done to follow up on these relationships in larger cohorts [49] to determine what wider role metabolic status at birth may influence future allergy risk.

## Supporting information

S1 Fig PCA plot of all data together. PC1 explains 72.5% of total variation. Data points are coloured according to farmer (1) or non-farmer (0) status. There are no clear analysis-related trends in the data.
(DOCX)

S2 Fig Box plots of the cord blood metabolites that differed (p<0.05) at birth between children born via vaginal birth and children born via caesarean section. Children born via vaginal birth are represented by the dark grey boxes and children born via caesarean section by the light grey boxes.
(DOCX)

S3 Fig Box plots of the cord blood metabolites that differed (p<0.05) at birth between children with siblings and children without siblings at birth. Children without siblings are represented by the dark grey boxes and children with siblings by the light grey boxes.
(DOCX)

S4 Fig Box plots of the cord blood metabolites that differed (p<0.05) between sexes. Females are represented by the dark grey boxes and males by the light grey boxes.
(DOCX)

S5 Fig Box plots of the cord blood metabolites that differed (p<0.05) at birth between non-allergic children and children diagnosed with allergy at 18 months. Non-allergic children are represented by the dark grey boxes and allergic children by the light grey boxes.
(DOCX)

S6 Fig Box plots of the cord blood metabolites that differed (p<0.05) at birth between non-allergic children and children diagnosed with allergy at 3 years. Non-allergic children are represented by the dark grey boxes and allergic children by the light grey boxes.
(DOCX)

S7 Fig Box plots of the cord blood metabolites that differed (p<0.05) at birth between non-allergic children and children diagnosed with allergy at 8 years old. Non-allergic children are represented by the dark grey boxes and allergic children by the light grey boxes.
(DOCX)

S1 Table Distribution of types of allergy among allergic children followed up at 18 months, 3 and 8 years. (DOCX)

S2 Table Correlation (Spearman’s rho) between metabolites associated with allergy development and maternal diet. (PDF)
