# Supplementary material for: Umbilical cord blood metabolome differs in relation to delivery mode, birth order and sex, maternal diet and possibly future allergy development in rural children
Source: PLoS One. 2021 Jan 25;16(1):e0242978. doi: 10.1371/journal.pone.0242978 (PMC7833224; doi:10.1371/journal.pone.0242978)
Supplement: S1 Fig — PC1 explains 72.5% of total variation. Data points are coloured according to farmer (1) or non-farmer (0) status. There are no clear analysis-related trends in the data. (DOCX) [file pone.0242978.s001.docx]

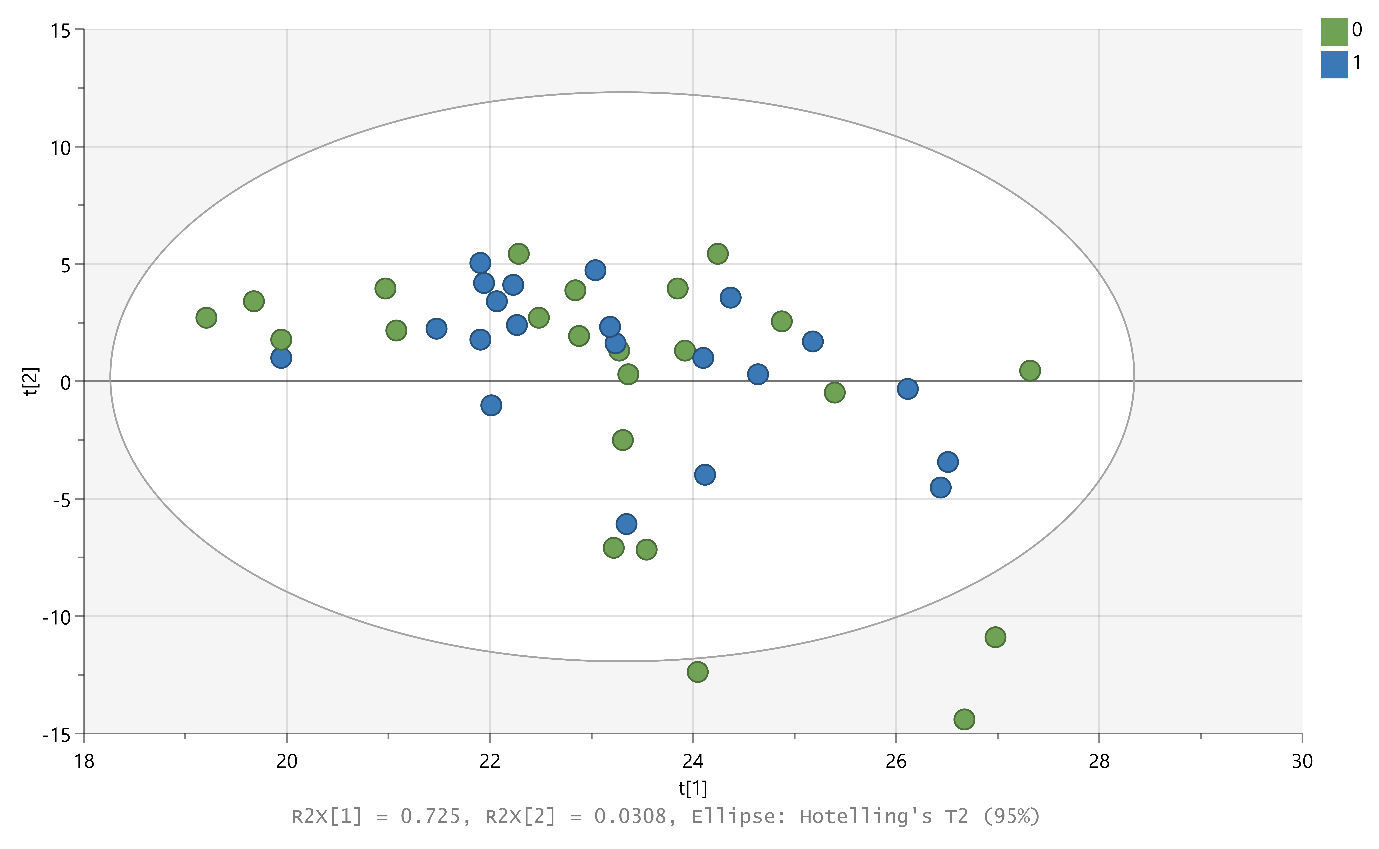


**Supplementary figure 1:** PCA plot of all data together. PC1 explains 72.5 % of total variation. Data points are coloured according to farmer (1) or non-farmer (0) status. There are no clear analysis-related trends in the data.
