# Supplementary material for: Umbilical cord blood metabolome differs in relation to delivery mode, birth order and sex, maternal diet and possibly future allergy development in rural children
Source: PLoS One. 2021 Jan 25;16(1):e0242978. doi: 10.1371/journal.pone.0242978 (PMC7833224; doi:10.1371/journal.pone.0242978)
Supplement: S2 Fig — Children born via vaginal birth are represented by the dark grey boxes and children born via caesarean section by the light grey boxes. (DOCX) [file pone.0242978.s002.docx]

**Supplementary Figure 2:** Box plots of the cord blood metabolites that differed (p<0.05) at birth between children born via vaginal birth and children born via caesarean section. Children born via vaginal birth are represented by the dark grey boxes and children born via caesarean section by the light grey boxes.


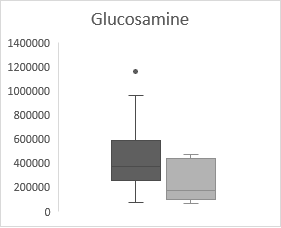

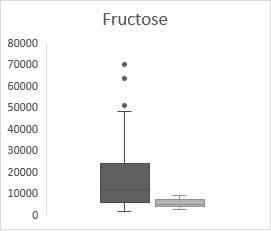

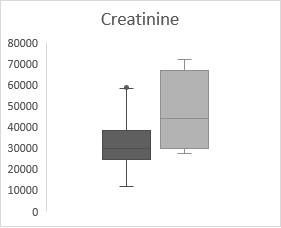

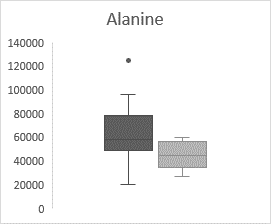


P=0.043

P=0.027

P=0.030

P=0.025

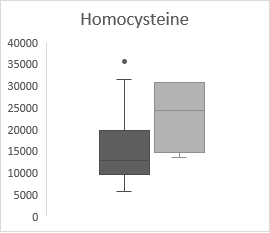

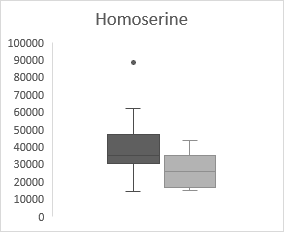

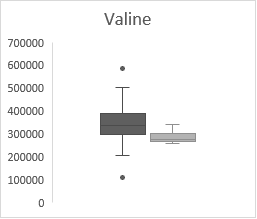

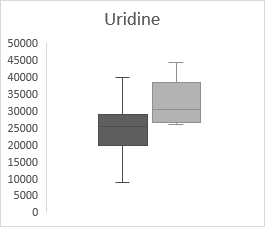

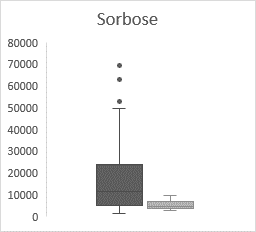

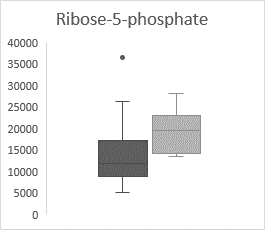

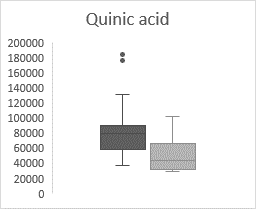

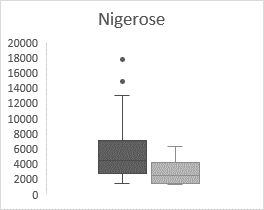

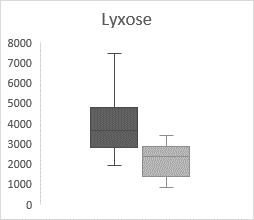

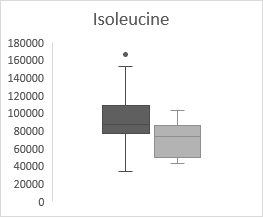


P=0.036

P=0.030

P=0.030

P=0.015

P=0.022

P=0.027

P=0.013

P=0.030

P=0.002

P=0.043
