# Supplementary material for: Umbilical cord blood metabolome differs in relation to delivery mode, birth order and sex, maternal diet and possibly future allergy development in rural children
Source: PLoS One. 2021 Jan 25;16(1):e0242978. doi: 10.1371/journal.pone.0242978 (PMC7833224; doi:10.1371/journal.pone.0242978)
Supplement: S3 Fig — Children without siblings are represented by the dark grey boxes and children with siblings by the light grey boxes. (DOCX) [file pone.0242978.s003.docx]

**Supplementary Figure 3:** Box plots of the cord blood metabolites that differed (p<0.05) at birth between children with siblings and children without siblings at birth. Children without siblings are represented by the dark grey boxes and children with siblings by the light grey boxes.


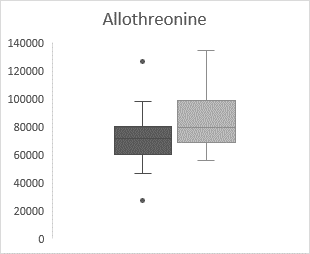

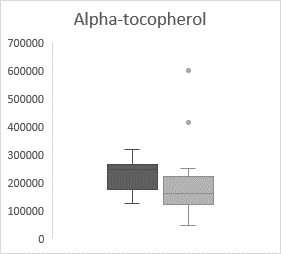

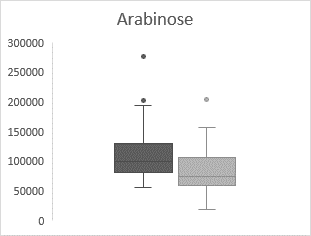

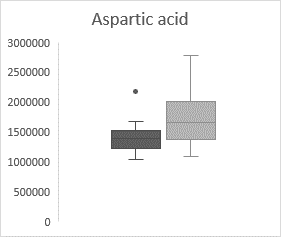

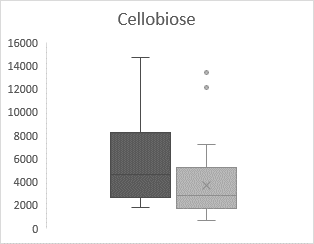

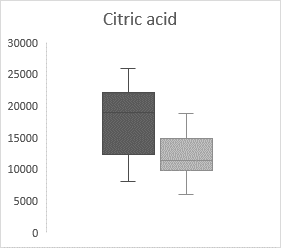

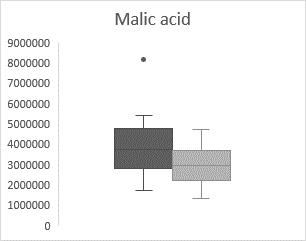

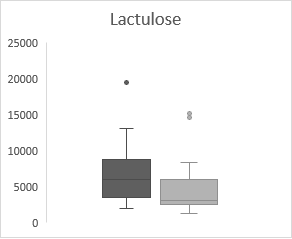

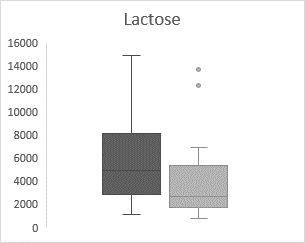

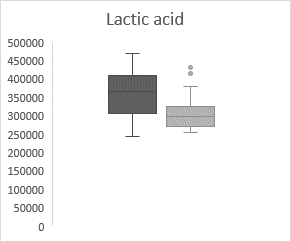

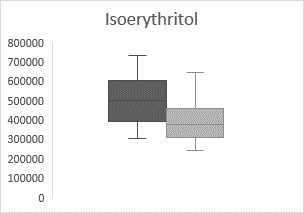

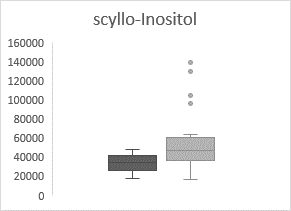

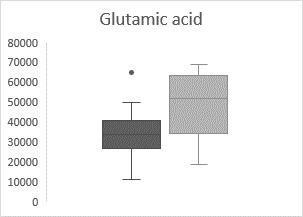

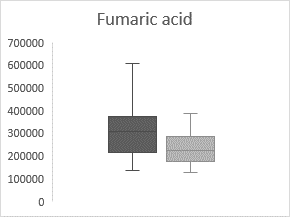

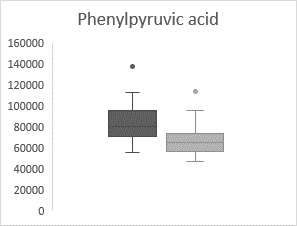

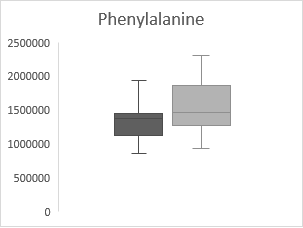

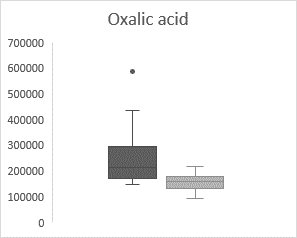

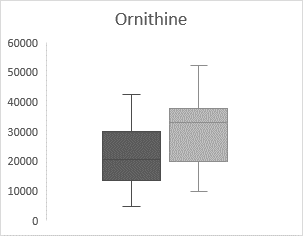

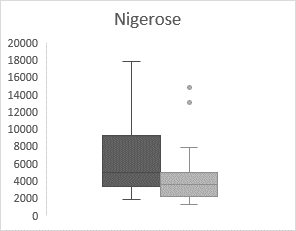

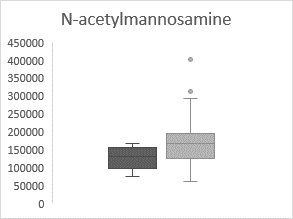

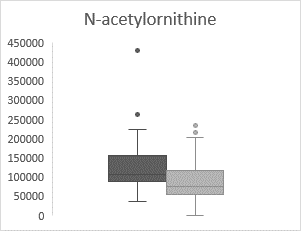

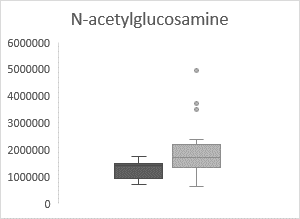

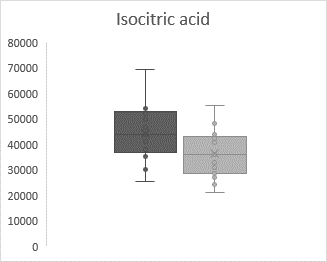

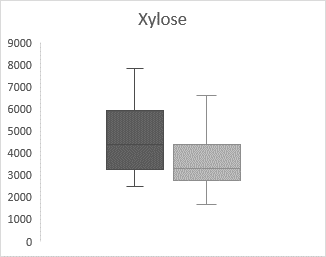

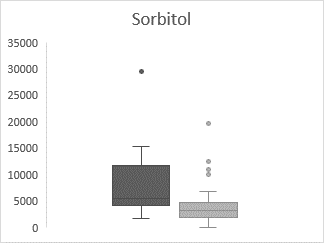

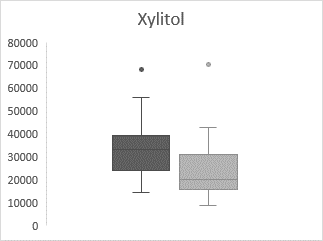


P=0.023

P=0.007vv

P=0.028

P=0.045

P=0.001

P=0.019

P=0.016

P=0.043

P=0.030

P=0.003

P=0.004

P=0.001

P=0.008

P=0.014

P=0.002

P=0.043

P=0.000

P=0.015

P=0.018

P=0.020

P=0.008

P=0.002

P=0.023

P=0.014

P=0.032

P=0.003
