# Supplementary material for: Umbilical cord blood metabolome differs in relation to delivery mode, birth order and sex, maternal diet and possibly future allergy development in rural children
Source: PLoS One. 2021 Jan 25;16(1):e0242978. doi: 10.1371/journal.pone.0242978 (PMC7833224; doi:10.1371/journal.pone.0242978)
Supplement: S4 Fig — Females are represented by the dark grey boxes and males by the light grey boxes. (DOCX) [file pone.0242978.s004.docx]

**Supplementary Figure 4:** Box plots of the cord blood metabolites that differed (p<0.05) between sexes. Females are represented by the dark grey boxes and males by the light grey boxes.


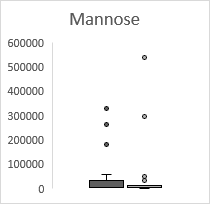

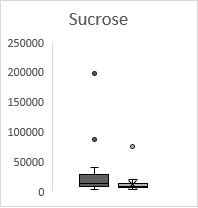

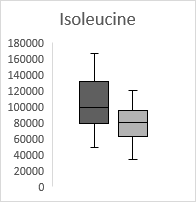

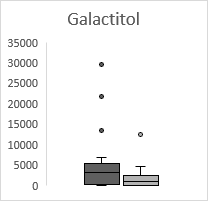

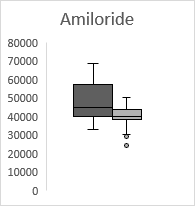

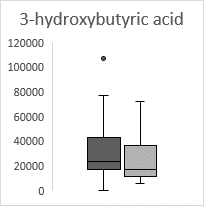

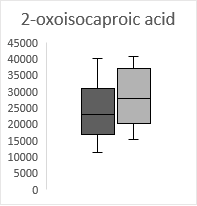


P=0.031

P=0.040

P=0.042

P=0.010

P=0.013

P=0.036

P=0.009

P=0.032

P=0.045


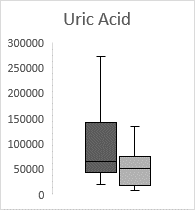

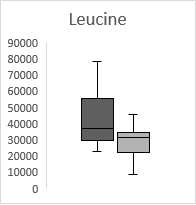

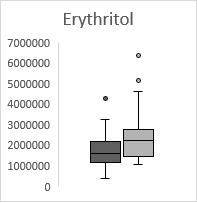


P=0.033
