# Supplementary material for: Umbilical cord blood metabolome differs in relation to delivery mode, birth order and sex, maternal diet and possibly future allergy development in rural children
Source: PLoS One. 2021 Jan 25;16(1):e0242978. doi: 10.1371/journal.pone.0242978 (PMC7833224; doi:10.1371/journal.pone.0242978)
Supplement: S5 Fig — Non-allergic children are represented by the dark grey boxes and allergic children by the light grey boxes. (DOCX) [file pone.0242978.s005.docx]

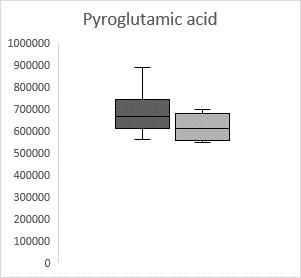
**Supplementary Figure 5:** Box plots of the cord blood metabolites that differed (p<0.05) at birth between non-allergic children and children diagnosed with allergy at 18 months. Non-allergic children are represented by the dark grey boxes and allergic children by the light grey boxes.

P=0.041

P=0.026

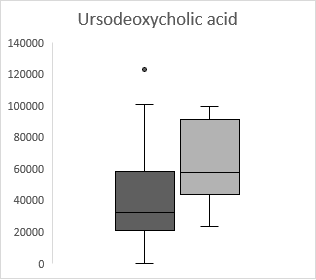

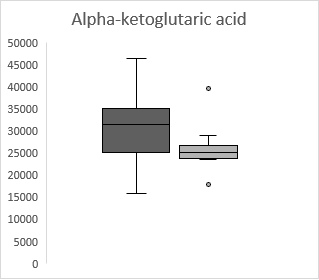


P=0.021
