# Supplementary material for: Umbilical cord blood metabolome differs in relation to delivery mode, birth order and sex, maternal diet and possibly future allergy development in rural children
Source: PLoS One. 2021 Jan 25;16(1):e0242978. doi: 10.1371/journal.pone.0242978 (PMC7833224; doi:10.1371/journal.pone.0242978)
Supplement: S1 Table — (DOCX) [file pone.0242978.s008.docx]

**Supplementary table 1: Distribution of types of allergy among allergic children followed up at 18 and 36 months, and 8 years.**

| **Child number** | **Allergy at 18 months** | **Allergy at 3 years** | **Allergy at 8 years** |
| --- | --- | --- | --- |
| **1** | ARC | ARC, Asthma | Asthma, ARC, Eczema |
| **2** | Asthma | Asthma | Asthma |
| **3** | Eczema | Eczema | Eczema |
| **4** | Eczema | Eczema | Eczema |
| **5** | Eczema | Food allergy | Healthy |
| **6** | Eczema, Asthma | Eczema | Healthy |
| **7** | Eczema, Asthma | Healthy | Asthma |
| **8** | Eczema, Asthma | Healthy | Healthy |
| **9** | Eczema, Asthma | Healthy | Healthy |
| **10** | Eczema | Healthy | No follow-up |
| **11** | Eczema | Healthy | Eczema |
| **12** | Eczema | Healthy | Healthy |
| **13** | Eczema | Healthy | Healthy |
| **14** | Food allergy, Eczema | Food allergy, Eczema | Healthy |
| **15** | Food allergy, ARC | No follow-up | No follow-up |
| **16** | Healthy | Eczema, Asthma | No follow-up |
| **17** | Healthy | Eczema | ARC |
| **18** | Healthy | Eczema | Healthy |
| **19** | Healthy | Asthma | No follow-up |
| **20** | Healthy | Healthy | Eczema |
| **21** | Healthy | Healthy | Asthma |
| **22** | Healthy | Healthy | ARC |

ARC: allergic rhinoconjunctivitis
