# Supplementary material for: Umbilical cord blood metabolome differs in relation to delivery mode, birth order and sex, maternal diet and possibly future allergy development in rural children
Source: PLoS One. 2021 Jan 25;16(1):e0242978. doi: 10.1371/journal.pone.0242978 (PMC7833224; doi:10.1371/journal.pone.0242978)
Supplement: S2 Table — (PDF) [file pone.0242978.s009.pdf]

Supplementary table 2: Correlation (Spearman's rho) between metabolites associated with allergy development and maternal diet.

	Spearman's rho	p
**Allergy at any time point**		
Opthalmic acid		
Wholegrain crispbread	-0.52	0.001
Ursodeoxycholic acid		
No correlated foods		
δ-tocopherol		
Butter in cooking	0.4	0.01
Glyceric acid		
No correlated foods		
Lactose		
Rapeseed/canola oil	0.47	0.004
Low-fat dressings and mayonnaise	0.46	0.005
White bread	0.43	0.009
Bacon and other cured pork	0.4	0.02
Cellobiose		
Low-fat dressings and mayonnaise	-0.5	0.002
Fermented milk	-0.47	0.004
Sorbitol		
No correlated foods		
Nigerose		
Low-fat dressings and mayonnaise	-0.5	0.002
White bread	-0.43	0.009
Bacon and other cured pork	0.44	0.02
**Allergy at 18 months**		
Ursodeoxycholic acid		
No correlated foods		
Pyroglutamic acid		
Salad dressing and mayonnaise	0.46	0.005
Wholegrain crispbread	-0.52	0.001
α-ketoglutaric acid		
Cabbage and lettuce	-0.41	0.02
Minced/ground meat	0.47	0.005
**Allergy at 3 years**		
Oxoisocaproic acid		
Butter on sandwiches	0.42	0.01
Margarine in cooking	-0.52	0.001
Low fat milk	-0.4	0.01
Uridine		
Banana	0.4	0.01
Tomato and cucumber	0.41	0.01
Cheese	0.44	0.005
Benzoic acid		
Porridge	0.41	0.01
Bacon and other cured pork	-0.43	0.008

Pickled herring	0.45	0.006
Coffee	-0.49	0.002
Putrescine		
No correlated food		
α-ketoglutaric acid		
Cabbage and lettuce	-0.41	0.02
Minced/ground meat	0.47	0.005
Isocitric acid		
White bread	-0.44	0.008
Popcorn and other snacks	-0.41	0.01
**Allergy at 8 years**		
E-Octadecenoic acid		
Mixed or soya oil in cooking	0.4	0.01
Porridge	0.47	0.004
Cabbage and lettuce	-0.41	0.02
Root vegetables	0.5	0.002
Pasta and rice salads	-0.54	0.001
Soft drinks	-0.4	0.01
Coffee	-0.54	0.001
Z-Octadecenoic acid		
Mixed or soya oil in cooking	0.5	0.002
Root vegetables	0.42	0.007
Pasta and rice salads	-0.48	0.003
Coffee	-0.58	0.001
Aspartic acid		
Boiled and baked potatoes	0.42	0.01
Lean sausage meals	0.42	0.01
Buns and crisp rolls	-0.43	0.008
Cholic acid		
Pasta	0.41	0.01
Hydroxyphenylacetic acid		
Olive oil in cooking	-0.42	0.01
Margarine	0.44	0.005
Rapeseed/canola oil	-0.59	<0.001
Hexadecanoic acid		
Mixed or soya oil in cooking	0.42	0.01
Coffee	-0.52	0.001
N-acetylornithine		
Green salad with cheese and ham	0.44	0.008
Added sugar and honey	0.47	0.004
Uric acid		
Margarine in cooking	0.44	0.007
